# Competition and Cooperation among Relational Memory Representations

**DOI:** 10.1371/journal.pone.0143832

**Published:** 2015-11-30

**Authors:** Hillary Schwarb, Patrick D. Watson, Kelsey Campbell, Christopher L. Shander, Jim M. Monti, Gillian E. Cooke, Jane X. Wang, Arthur F. Kramer, Neal J. Cohen

**Affiliations:** 1 University of Illinois, Beckman Institute for Advanced Science and Technology, Urbana, IL, United States of America; 2 Northwestern University Feinberg School of Medicine, Department of Medical Social Sciences, Chicago, IL, United States of America; University of Lethbridge, CANADA

## Abstract

Mnemonic processing engages multiple systems that cooperate and compete to support task performance. Exploring these systems’ interaction requires memory tasks that produce rich data with multiple patterns of performance sensitive to different processing sub-components. Here we present a novel context-dependent relational memory paradigm designed to engage multiple learning and memory systems. In this task, participants learned unique face-room associations in two distinct contexts (i.e., different colored buildings). Faces occupied rooms as determined by an implicit gender-by-side rule structure (e.g., male faces on the left and female faces on the right) and all faces were seen in both contexts. In two experiments, we use behavioral and eye-tracking measures to investigate interactions among different memory representations in both younger and older adult populations; furthermore we link these representations to volumetric variations in hippocampus and ventromedial PFC among older adults. Overall, performance was very accurate. Successful face placement into a studied room systematically varied with hippocampal volume. Selecting the studied room in the wrong context was the most typical error. The proportion of these errors to correct responses positively correlated with ventromedial prefrontal volume. This novel task provides a powerful tool for investigating both the unique and interacting contributions of these systems in support of relational memory.

## Introduction

Memory performance depends on differentiable memory systems that process different information [1–3] and rely on different neural substrates [4–8]. The binding-in-context model [9,10] emphasizes a differentiation between memory systems sensitive to specific associations [11,12] and general contexts [13,14]. These different information sources act in a broader relational memory system [6,15,16] that binds arbitrary relations between specific items and broader contextual information.

Under most conditions, memory networks tuned to contextual rules and individual associations interact, compete, and cooperate seamlessly to produce patterns of performance appropriate to the task at hand. Thus, clearly distinguishing between different sources of information is an empirical challenge that requires memory tasks capable of assessing how these systems compete and cooperate during memory performance. For example, the *exclude recognition* approach [17] asks participants to remember many items (e.g., words) and distinguish between the contextual sources of those items (the particular list on which they appeared). Resulting data suggest a dynamic interaction between memory sources, wherein contextual information can either refine or guide the search of item memory [14,18–20] or induce competition and interference at retrieval [21,22]. Similarly, segmentation approaches [23] group stimuli into sets and measure the frequency of errors within and between these sets. The general finding, that items are more likely to be confused within than between sets, demonstrates that the segmentation boundaries themselves form an important part of the representation. Furthermore, in paradigms that allow participants to freely reconstruct the pattern of stimuli that most closely matches their remembered representation, systemic errors are often reflective of schematic prior knowledge [24,25] or improperly configured studied information [26,27]. Such errors can serve as a useful tool in understanding different sources of interference contributing to a memory representation and more sensitive behavioral measures, such as eye-tracking, can reveal the influence of remembered, but implicit information [28].

Across all of these approaches, one common thread is that when memory errs, it often produces the “second best” response, and that this *approximately correct* answer reflects information processed by a memory system that is interacting with the dominant *correct-answer* producing system. Through sensitive behavioral measures and appropriate analyses of the full pattern of participants’ performance, it is possible to glean information about non-dominant memory systems and better understand the interaction of all the memory systems that contribute to task performance.

**Fig 1:**
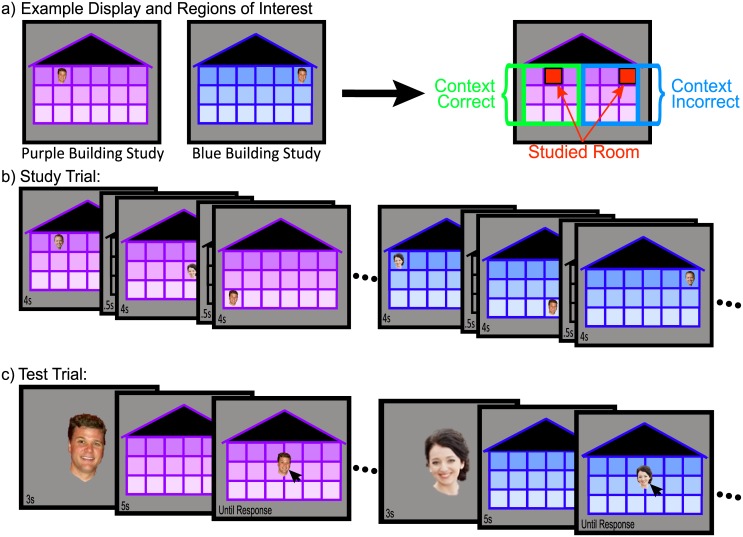
Stimuli and design in the novel context-dependent relational memory test. (a) General display information and resulting regions of interest. (b) Study phase design and timing information. (c) Test phase design and timing information.

In the current study, we explore the dynamics of competition and cooperation between sources of associative and contextual information as well as the memory systems supporting these processes. We introduce a novel memory paradigm (Fig 1) that provides participants with two sources of information: specific item-location associations, and general contextual rules. Participants studied a set of faces that appeared in specific rooms of multi-room building contexts that differ by color (one building is blue, one purple). Each face appeared in a unique room in each building (all faces were studied in both buildings). Face-room pairs were assigned using an underlying gender-by-side rule structure (e.g., female faces on the left in the blue building and on the right in the purple). At recall, participants were asked to place faces back in the room in which they were studied. Each face was tested in both building contexts.

Because the number of rooms is large, the “best” answer (i.e., placing a face in the *studied*, *context-correct* (CC) room) places large demands on memory encoding. However, even if participants fail to retrieve this best answer, retrieving some information can still yield a “second best” response. Participants might place a face in a room in which it was *studied*, but in the alternative context, choosing a remembered, but *context-incorrect* (CI), association. Or participants might choose to place the face in a way that obeys the context-sensitive gender-by-side rule, but in an unstudied, incorrect location. This trade-off between *CC* and *studied* associations speaks to the relative strength of these sources of information active in the participant’s representation. In the two experiments presented here, employing data from behavioral responses, eye tracking, and structural MRI, we attempt to 1) modulate the relative strength of these sources of information, 2) dissociate their contributions, and 3) identify brain structures that might differentially contribute to successful performance on our novel memory task.

Based on previous literature, we hypothesized that two neural regions would be directly involved in successful performance on this task: the hippocampus and the ventromedial prefrontal cortex (vmPFC). The hippocampus has been implicated in countless other relational-memory paradigms (e.g., [27,29–34]). It is well established that patients with hippocampal damage are impaired in remembering the spatial arrangements of objects (e.g., [27,35]), face-scene pairs (e.g., [36]), which items appeared together (e.g., [33]), and object-location bindings (e.g., [37]) even at very short delays. Together these data support a generalized mechanism in which the hippocampus rapidly forms associations and binds these elements into relational representations [38]. Therefore, in the current study we hypothesized that the hippocampal memory system would be important for binding of arbitrary relations among items, specifically binding faces to studied rooms (e.g., [8,25]).

While the hippocampus is essential to binding disparate information into a coherent memory representation, the vmPFC has recently emerged as another brain region with a related, specialized function. Both the hippocampus and vmPFC are critically involved in the inferential use of associative memories (e.g., [39]) such that prior experiences are reactivated in similar situations allowing integration of new information with overlapping previously learned information [40]. The vmPFC receives direct inputs from the hippocampus [41] and this pathway supports memory consolidation and the strategic control of memory (for review see [42]). Thus, while the hippocampus links elements to form new memory associations, when new information overlaps with those learned associations, the vmPFC reconciles the conflicting representation by incorporating the new information into the existing representation [42]. Thus while the hippocampus binds elements such as A > B and B > C, the vmPFC is necessary to integrate those elements and update the representation to indicate that A > B > C. Indeed, patients with vmPFC damage are severely impaired on tests of transitive inference [43]. Therefore, in the current task we hypothesized that the vmPFC system would be more important for associating multiple representations that share a common feature [40,43]; that is, the vmPFC would identify that a given face was studied in, for example, room 4 of one building and room 12 of the other and combine these two separate representations into a single three-part representation.

Additionally, both hippocampal and prefrontal regions have been implicated in successful memory performance in many imaging studies (for reviews see [44,45]), and this task provides a possible means of dissociating their contribution in a single paradigm. Further, it is well established that both hippocampal volume (e.g., [46]) and frontal function [47,48] decline with aging, with associated behavioral changes. Because this task targets differential contributions of these memory regions, we hypothesized that integrity of these brain substrates in a cohort of older adults might be related to different types of associative memory performance.

Across both experiments, our goal was to demonstrate the consequences of systematically varying the relative contribution of associative and contextual memory systems. Reinforcing studied associations ought to increase overall accuracy, but at the cost of also increasing CI-studied responses (Experiment 1); reinforcing contextual rules also ought to increase accuracy encouraging more CC-studied responses (Experiment 2). The shifting of participants’ preference for different responses informs us about the competition and cooperation of their underlying representations. By using multiple, non-dichotomous, and converging methods (i.e., unconstrained response, eye-tracking, and structural brain measures) we can unpack how these memory systems interact, and move toward predicting specific patterns of responses.

## Materials and Methods

### Experiment 1: Competition between contextual and associative information

#### Participants

Forty-nine participants (ages 18–28; 17 males) from the University of Illinois Urbana-Champaign participated in this study. Nine participants were excluded from the analysis because their eye position could not be calibrated reliably. One block was excluded from a 10^th^ participant due to a data logging error. Participants were paid $8 per hour for their participation.

#### Apparatus

Eye positions were recorded at a rate of 500Hz using an Eyelink 1000 eye-tracking system (SR Research, Ontario, Canada). Participants were positioned in a chin rest 60cm from the screen. All stimuli were presented on a 21” color monitor and data were collected using Presentation (Neurobehavioral Systems, http://nbs.neuro-bs.com) on a Windows-based computer.

#### Stimuli and Design

Two buildings, differing only in color, one purple and one blue, were constructed each with 3 rows (“floors”) of 6 rooms. There was a gradation in shade across the floors, as can be seen in Fig 1a and 1b. Each building was sized to 29.2 x 21.3° of visual angle and served as a unique context in which faces appeared. Twenty-four male and 24 female full-color face images were selected from our face database (see [49]). Versions of each face were sized to approximately 3.2° x 3.7° of visual angle (“small”; for the study phase and latter portion of the test phase) and approximately 6.9° x 7.9° of visual angle (“large”; for the initial portion of the test phase). Four male and four female faces were presented individually in each of six experimental blocks.

Each face appeared once in each building context, in one room on the left side of one building and one room on the right side of the other building. Possible face-room pairings were restricted so that a given face never appeared in adjacent rooms across building contexts. Male and female faces always appeared on opposite sides of a given building context, and on different sides for each building context (e.g., females on the left in one building and males on the left in the other). Counterbalancing across participants ensured that each room was occupied equally often and each face-room pairing was tested an equal number of times.

Each block was divided into a study phase and a test phase. During the study phase, participants learned where each of the eight “small” faces belonged in one building context before learning where those same faces belonged in the second building context. Face-room pairs were presented sequentially and each pair remained on the screen for 5s (500ms inter-trial-interval). On each trial, participants responded to the encoding question “On which floor is the face located?” with a button push. During the testing phase (Fig 1a), a “large” face was centrally presented for 3s and was then replaced by one of the (blue or purple) empty building contexts for 5s. Participants were encouraged to visually explore the building and decide where the face belonged. The “small” face then reappeared, and participants used the mouse to position the face into the appropriate room. All eight faces were tested in one building context before they were tested in the other building context.

Participants were randomly assigned to two groups. The Study1x group studied each face-room pair once before test. The Study3x group repeated the study phase three times prior to test. The order in which the different building contexts appeared, both at study and at test, was counterbalanced across participants; and the gender-by-side assignment (e.g., males on left in the blue building context) was also counterbalanced across participants.

#### Procedure

After providing written informed consent (both the study and consent procedure were approved by the University of Illinois Urbana-Champaign Institutional Review Board; IRB Approval #01097), instructions were given and participants then completed 12 practice study trials and 12 practice test trials. Practice trials were identical to experimental trials. Following practice, a chin rest was positioned directly in front of the monitor, and eye position was calibrated using a 3x3 array of fixation crosses. Full calibration was performed prior to each study phase and a calibration stability check was performed prior to each testing phase. Eye position and fixation duration were recorded during test trials only. Participants completed six blocks of trials with self-timed breaks after each testing phase. The procedure was identical for the two groups except for the critical study-phase manipulation (i.e. one vs. three presentations).

#### Statistical Analyses

Data were categorized into two main regions-of-interest (Fig 1a): CC rooms (nine rooms on the side of the building corresponding to a correct response based on the contextual-rule structure) and CI rooms (nine rooms on the side of the building corresponding to an incorrect response). Data were further subdivided into four subregions-of-interest: CC-studied room (the single room in which a face had been studied in a given building), CC-unstudied rooms (the eight remaining rooms on the CC side of the building), CI-studied room (the single room in which the face had been studied, but in the other building), and CI-unstudied rooms (the remaining eight CI rooms). Specific analyses are detailed in the Results section. Huynh-Feldt corrected statistics are reported where appropriate.

### Experiment 2: Competition within and between multiple sources of information

#### Participants

Twenty-nine young adult participants (ages 19–32; 7 males) from the University of Illinois participated in this study. Eight participants were excluded from the analysis because their eye position could not be calibrated reliably, and a ninth participant was excluded due to a data logging error. Participants were paid $8 per hour.

Twenty-two older adult participants (ages 61–85; 7 males) from the Champaign-Urbana community participated in this study. Two participants were excluded from the analysis because their eye position could not be calibrated reliably, and one block was excluded from a third participant due to a data logging error. All participants had normal to corrected-to-normal vision. Participants were paid $10 per hour.

#### Apparatus

The apparatus was identical to that used in Experiment 1.

#### Stimuli and Design

**Fig 2:**
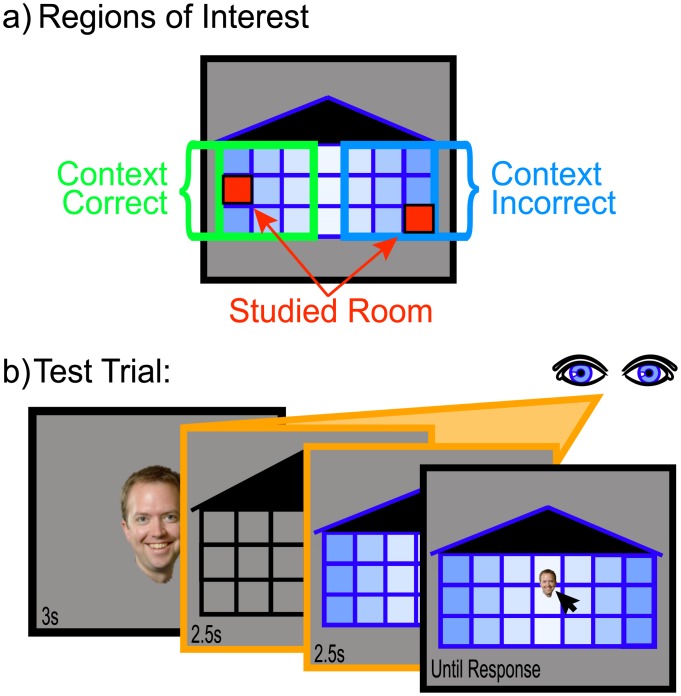
Design modifications used in Experiment 2. (a) General display information and resulting regions of interest. (b) Test phase design and timing information. Orange boxes indicate screens for which eye-tracking data was collected.

The stimuli were identical to Experiment 1 with four exceptions: (1) An additional column of rooms was added to each building such that there were 3 rows (“floors”) of 7 rooms each and the entire building subtended 29.5° x 16.9° of visual angle (Fig 2a). (2) There was a gradation in shade across columns that radiated out from the center column. (3) During each study trial, participants answered the encoding question, “On which side of the building does the face appear?” They indicated left or right with a button push. (4) Thirty male and 30 female full-color face images were selected from our face database (see [49]) and each face was sized to approximately 2.6° x 3.1° of visual angle (“small”; for the study phase and latter portion of the test phase) and approximately 6.9° x 7.9° of visual angle (“large”; for the initial portion of the test phase). As in Experiment 1, the order in which the different building contexts appeared was counterbalanced across participants; and the gender-by-side assignment (e.g., males on left in the blue building context) was also counterbalanced across participants. Counterbalancing across participants ensured that each room was occupied equally often and each face-room pairing was tested an equal number of times.

The design was similar to Experiment 1 with three modifications: (1) All participants saw each face-room pair three times prior to test (as in Experiment 1’s Study3x condition) and faces never appeared in the center column during study. (2) During the testing phase, a “large” face was centrally presented for 3s and was then replaced by an uncolored contextually ambiguous (i.e., gray) building. After 2.5s, the context was revealed by replacing the gray building with one of the two colored buildings that remained on the screen for an additional 2.5s. The “small” face then reappeared and participants used the mouse to position the face into the appropriate room (Fig 2b). (3) All eight studied faces as well as four novel faces (two male and two female) were tested once in each building. Participants were instructed to, “Place any new face where you think it fits best.”

#### Procedure

The procedure was identical to that of Experiment 1 with three exceptions: (1) Full calibration was performed before each study and each test phase and eye position and fixation duration was recorded during both study and test trials. (2) Each participant completed five study and five testing phases. (3) Structural MRI scans were collected for all older adults.

#### Image Acquisition

Images were collected on a Siemens Magnetom Trio 3T whole body MRI scanner. A standard 12-channel birdcage head coil was used and head motion was restricted with foam padding. High-resolution 3D MPRAGE (TI = 900ms; flip angle = 9°; .9 mm isotropic voxels) structural images were acquired in the sagittal plane. Structural scans were acquired 8–25 months (average = 16.5 months) prior to behavioral data collection.

#### Volumetric analysis

Automatic segmentation of the hippocampus, striatum (caudate and putamen), and vmPFC was performed using Freesurfer (v 5.3; details about the subcortical segmentation process have been described in [50]). Freesurfer was also used to calculate automated measures of intracranial volume (ICV; see [47] for detailed method) comparable to manual tracing and this value was used to correct cortical and subcortical volumes for overall head size. By regressing each ROI volume onto ICV, a slope (*b*) was obtained for the relationship between ROI and ICV. The resulting slope was used to normalize each ROI for head size (normalized volume = raw brain volume–*b*(ICV-mean ICV); [46,51,52]). To improve correction, mean ICV was calculated from a larger group of 76 older adults, 20 of which were the participants in the current study.

#### Statistical analyses

All responses and fixations made to the center column were excluded from all analyses. Regions-of-interest and statistical analyses were identical to Experiment 1 except that partial correlation analyses were also performed relating older adults’ regional brain volumes to task performance accounting for age.

## Results

### Experiment 1: Competition between contextual and associative information

In Experiment 1, participants were randomly assigned to two groups. One group studied face-room pairs once before test (Study1x), while the other studied each pair three times before test (Study3x). This manipulation resulted in between group differences in the strength of learned associative information.

#### Behavioral Performance

Participants in the Study1x group placed faces on the CC side of the building 75.9% of the time and on the CI side only 23.1% of the time. Participants in the Study3x group placed faces on the CC side of the building 72.1% and the CI side 27.9% of the time. A Side (CC, CI) x Group (Study1x, Study3x) repeated measures ANOVA revealed a main effect of side, *F*(1,38) = 119.15, *p <* .001, *η* ^2^ = .758, indicating that participants in both groups preferred the CC side of the building. The interaction was not significant, *F*(1,38) = 0.77, *p =* .386, *η* ^2^ = .020, indicating that this CC preference was similar for both groups.

**Fig 3:**
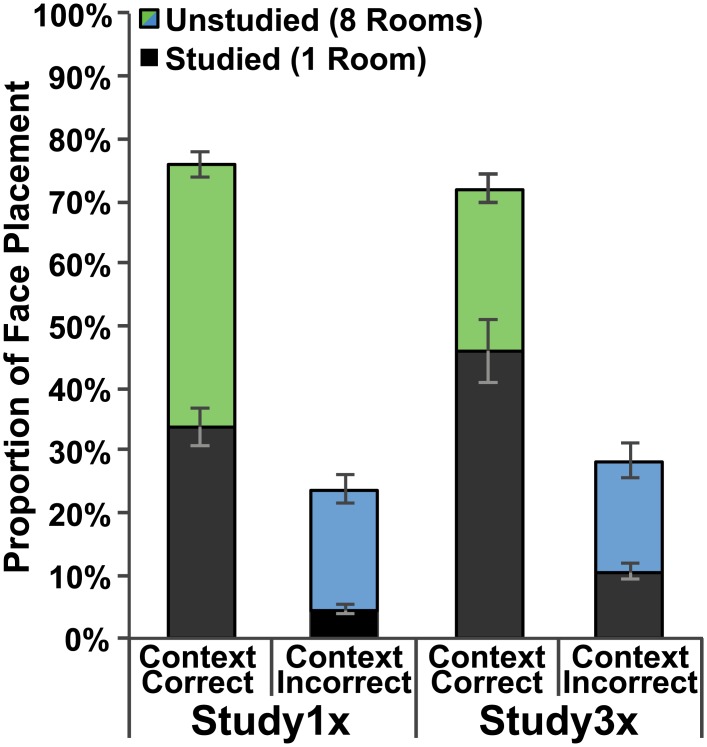
Behavioral performance. Proportion of face placement to rooms on the correct (green) and incorrect (blue) side of the building. Dark bars indicate face placements to the studied room on a given side whereas colored (green or blue) bars indicate face placements to any of the remaining eight unstudied rooms. Data are presented for both the Study1x and Study3x groups and standard error bars are shown.

Furthermore, participants in the Study1x group made 33.8% of their total placements to the CC-studied room and 4.6% to the CI-studied room; this difference was significant *t*(19) = 9.45, *p <* .001 (Fig 3). Participants in the Study3x group made 46.1% of their total placements to the CC-studied room and 10.7% to the CI-studied room; this difference was also significant *t*(19) = 5.87, *p <* .001 (Fig 3). These data show that participants in both groups are more likely to choose the CC-studied room compared to the CI-studied room. However, independent samples *t*-tests indicated that people in the Study3x group made both more CC-studied responses, *t*(38) = -2.08, *p =* .045, and more CI-studied responses, *t*(38) = -4.03, *p <* .001, than participants in the Study1x group indicating that with additional study opportunities, participants make more CC-studied and CI-studied room placements.

#### Studied Room Preference

**Fig 4:**
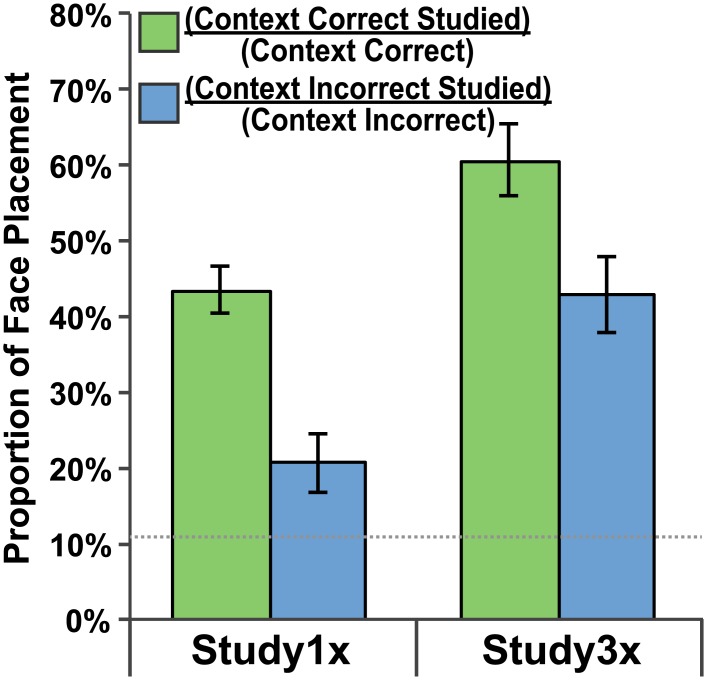
Studied room preference. Preference for face placements to the studied room as a proportion of all face placements to a given side of the building. Green bars indicate a preference for the studied room on the context-correct side of the building. Blue bars indicate a preference for the studied room on the context-incorrect side of the building. Data are presented for both the Study1x and Study3x groups and standard error bars are shown. The dotted gray line indicates chance-level performance.

To determine the preference for the studied room over other rooms on a given side of the building, the proportion of studied room placements to total placements was computed for each side of the building. Study1x participants preferred the studied room 43.5% of the time on the CC side and 20.6% of the time on the CI side of the building (Fig 4). Study3x participants preferred the studied room 60.6% of the time on the CC side and 39.8% of the time on the CI side of the building (Fig 4). One-sample *t*-tests indicated that these studied-room preferences were greater than chance (11.1%; *p <* .02 in all cases). These data suggest that participants showed a preference for the studied room even when they made a CI response.

#### Rule learning

Rule learning was evaluated by identifying where participants put faces when they did not select either of the two studied rooms. As such, participants in the Study1x group made 69.9% and the Study3x group made 64.2% CC placements. One-sample *t*-tests comparing CC placement to chance (50%) revealed that participants in both groups were significantly more likely to make a CC placement than would be expected by chance (*t*>4, *p <* .002 in both cases). Imprecise memory representations resulting in studied*-*adjacent responding could also produce this pattern of results. However, when participants made a studied*-*adjacent response to a boundary target (i.e., those rooms in the third and fourth columns of the building), one-sample *t*-tests (compared to 50%) revealed that they were again significantly more likely to make CC placements (Study1x: *t*(19) = 4.4, *p <* .001; Study3x: *t*(19) = 2.7, *p =* .015). These data suggest that in the absence of specific learned associations, participants in both groups relied on the contextual rule-structure to respond.

#### Eye Tracking Data

Proportion of viewing to rooms in which faces were studied was calculated for each participant. Eye tracking data showed similar trends as behavioral data in this experiment and do not provide additional information. These data are, therefore, not reported.

#### Summary

Thus in Experiment 1, participants consistently preferred the CC side of the building to make their responses. Additionally, the studied room was chosen for face placement significantly more than would be expected by chance and participants were more likely to choose the studied room on the CC side than the CI side. The Study3x group made significantly more CC-studied responses and CI-studied responses than the Study1x. These data suggest that with additional study opportunities, the representation for the studied room on both sides of the building was strengthened providing very early evidence that participants learned to associate a single face with two different rooms. These data hint at the idea that perhaps a triadic room1-face-room2 representation has been formed [40,43] and that at the time of retrieval, the CC-studied room and CI-studied room may compete for representation. Competition between the two associated rooms could be resolved by applying knowledge of the underlying rule structure. This possibility was further explored in Experiment 2.

### Experiment 2: Competition within and between multiple sources of information

In Experiment 2, four specific modifications were made to the task in order to emphasize the contextual rule structure. (1) Each building was expanded to include seven columns. In order to emphasize the distinctions between the sides of each building context, there was a gradation in shade across the columns, as can be seen in Fig 1c. Faces were never assigned to the center column, making the “male side” and “female side” non-contiguous. (2) During study, the encoding question asked participants to indicate on which side of the building the face appeared, rather than about the floor as in Experiment 1. Emphasizing the underlying rule structure allowed us to more directly observe competition between associative and contextual sources of information. (3) At test, participants were first presented with a non-colored context*-*neutral version of the building (i.e., a gray building) before any contextual information appeared (Fig 1c). Eye movements recorded during this gray-building preview allowed us to examine competition within the relational memory system independent of contextual rule information. (4) Finally, unstudied novel faces were included at test as a pure measure of contextual-rule learning. To preview our findings, these manipulations appear to have emphasized the contextual-rule structure in Experiment 2. Participants made more CC placements and fewer CI placements than in the comparable (Study3x) condition in Experiment 1.

#### Behavioral Performance

Participants in the Young Adult group placed faces on the CC side of the building 83.7% and the CI side 16.3% of the time. Participants in the Older Adult group placed faces on the CC side of the building 60.7% of the time and on the CI side only 39.3% of the time. A Side (CC, CI) x Group (Young, Old) repeated measures ANOVA revealed a main effect of side, *F*(1,38) = 89.48, *p <* .001, *η* ^2^ = .702, indicating that participants in both groups preferred the CC side of the building. The interaction was also significant, *F*(1,38) = 23.96, *p =* .39, *η* ^2^ = .387, indicating that the preference for CC rooms was greater for younger compared to older adults.

**Fig 5:**
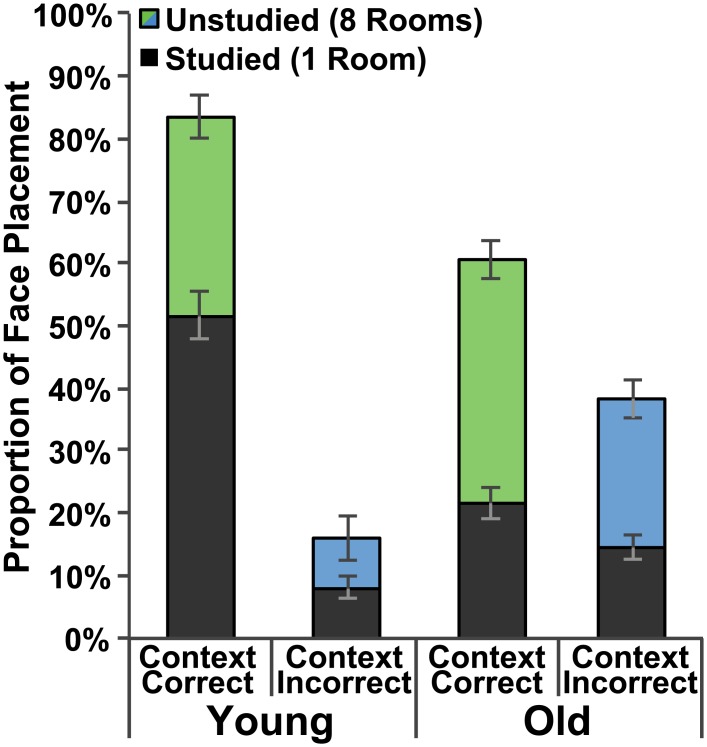
Behavioral performance. Proportion of face placement to rooms on the correct (green) and incorrect (blue) side of the building. Dark bars indicate face placements to the studied room on a given side whereas colored (green or blue) bars indicate face placements to any of the remaining eight unstudied rooms. Data are presented for both the Young Adult and Older Adult groups and standard error bars are shown.

Participants in both groups preferred the CC-studied to the CI-studied room. Young adults made 51.7% of their total placements to the CC-studied room and 8.1% to the CI-studied room; this difference was significant *t*(19) = 8.42, *p <* .001 (Fig 5). Older adults made 21.6% of their total placements to the CC-studied room and 14.6% to the CI-studied room; this difference was also significant *t*(19) = 3.06, *p =* .006 (Fig 5). A Trial Type (CC-studied, CI-studied) x Group (Young Adults, Old Adults) repeated measures ANOVA revealed a main effect of Trial Type, *F*(1,38) = 79.89, *p <* .001, *η* ^*2*^ = .68, indicating that both groups preferred the CC-studied room. The interaction, *F*(1,38) = 41.76, *p <* .001, *η* ^*2*^ = .52, was also significant with the CC-studied room preference being reduced for older adults. Finally, the main effect of Group, *F*(1,38) = 22.99, *p <* .001, *η* ^*2*^ = .38, was significant indicating that older adults made overall fewer CC-studied and CI-studied room placements than young adults.

#### Studied Room Preference

**Fig 6:**
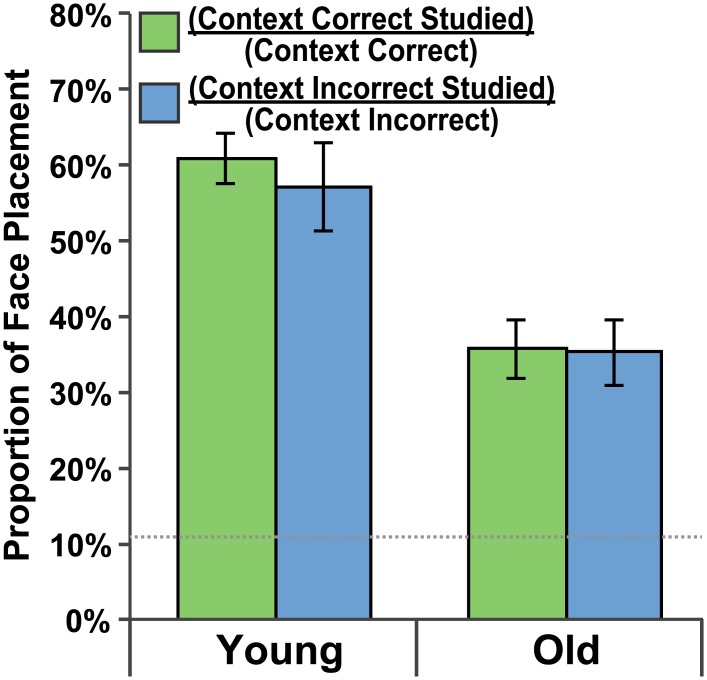
Studied room preference. Preference for face placements to the studied room as a proportion of all face placements to a given side of the building. Green bars indicate a preference for the studied room on the context-correct side of the building. Blue bars indicate a preference for the studied room on the context-incorrect side of the building. Data are presented for both the Young Adult and Older Adult groups and standard error bars are shown. The dotted gray line indicates chance-level performance.

Again, to determine the preference for the studied room over other rooms on a given side of the building, the proportion of studied room placements to total placements was computed for each side of the building. Young adult participants preferred the studied room 60.8% of the time on the CC side and 57.2% of the time on the CI side of the building (Fig 6). Older adult participants preferred the studied room 35.7% of the time on the CC side and 35.3% of the time on the CI side of the building (Fig 6). One-sample t-tests indicated that these studied-room preference were greater than chance (11.1%; *p <* .001 in all cases). A Side (CC, CI) x Group (Young, Old) repeated measures ANOVA was performed. Neither the main effect of Side, *F*(1,38) = .30, *p =* .589, *η* ^*2*^ = .008, nor the interaction, *F*(1,38) = .19, *p =* .67, *η* ^*2*^ = .005, was significant indicating that both groups preferred the studied room to all other rooms similarly on each side of the building. The main effect of Group, *F*(1,38) = 21.10, *p <* .001, *η* ^*2*^ = .36, was significant with the young adults showing a stronger preference for the studied room than older adults. Again these data suggest that participants showed a preference for the studied room even when they made a CI response.

#### Rule learning

Novel face placement was used as a metric for rule learning. If a participant placed a novel face on the CC side of the building, this suggests that the participant had learned the underlying gender-by-side rule structure. One young adult was removed from the analysis for only ever placing novel faces in the center column of the building; all trials were excluded from the analysis. Young adults placed novel faces on the CC side of the building 75.9% of the time; this was significantly different from chance (50%), *t*(18) = 5.5, *p <* .001. Older adults placed the novel face on the CC side of the building 59.3% of the time; this was also significantly different from chance, *t*(19) = 2.8, *p =* .011. Young adults placed novel faces on the CC side significantly more than older adults, *t*(37) = 2.9, *p =* .006. Thus while both groups showed evidence of rule learning, younger adults used the rule more frequently when placing novel faces.

#### Eye Tracking Data

Proportion of viewing to rooms in which faces were studied was calculated for each participant.

#### Effect of Repeated Study

Disproportionate selection of the CC-studied and CI-studied rooms at test suggests that participants learned to associate a single face with two rooms and these two possible target locations competed for selection. We can more directly test the existence of such triadic representations by evaluating looking behavior during study. Participants studied each face in each building context a total of three times. Young adults spent most of their time (68.5%) looking at the CC-studied room where the face was displayed on the screen. However, a Repetition (1, 2, 3) x Trial Type (CC-studied, CI-studied) ANOVA revealed a significant main effect of Repetition (*F*(2,38) = 22.8, *p <* .001, *η* ^*2*^ = .545), a significant main effect of Trial Type (*F*(1,19) = 439.1, *p <* .001, *η* ^*2*^ = .959), and a significant interaction (*F*(1.3,23.9) = 25.4, *p <* .001, *η* ^*2*^ = .572). This interaction indicates that across these multiple study repetitions, the proportion of viewing of the CC-studied room decreased and viewing of the CI-studied room increased despite the CI-studied room being an empty room, visually identical to the other 19 empty rooms. Increased viewing to the CI-studied room during study provides additional support for the formation of triadic room1-face-room2 memory representations. These data suggest that with each repetition participants learned that each face belonged to two rooms and they checked both during study. Older adults also spent most of their time (76.8%) looking at the CC-studied room and again the proportion of viewing to the CC-studied room decreased while viewing to the CI-studied room increased. The repeated measures ANOVA revealed a significant main effect of Repetition (*F*(1.6,30.8) = 7.4, *p =* .004, *η* ^*2*^ = .281), a significant main effect of Trial Type (*F*(1,19) = 604.3, *p <* .001, .970), and a significant interaction (*F*(1.2,23.3) = 8.2, *p =* .006, *η* ^*2*^ = .301). Thus, with each repetition older adults learned that each face belongs in two separate rooms (i.e., they form a triadic representation of the possible rooms associated with each face) and looked to both rooms during study. A Repetition (1, 2, 3) x Trial Type (CC-studied, CI-studied) x Group (Young Adults, Older Adults) showed a significant three-way interaction, *F*(2,76) = 6.1, *p =* .004, *η* ^*2*^ = .138, demonstrating that the effect was more pronounced for the young adults.

#### Viewing During the Ambiguous Context

In this experiment, each face was tested twice, once in each building. Young adults were able to keep track of which building had been tested and anticipate which would be tested the second time; as such the gray building period was only ambiguous the first time a face was tested. A Test Time (First, Second) x Trial Type (CC-studied, CI-studied) repeated measures ANOVA showed a significant interaction, *F*(1,19) = 5.8, *p =* .026, *η* ^*2*^ = .234, and for this reason, only first-test trials were included in the following analyses. Older adults did not show this pattern of viewing (non-significant interaction, *F*(1,19) = .4, *p =* .527, *η* ^*2*^ = .021) and all test trials are included here.

**Fig 7:**
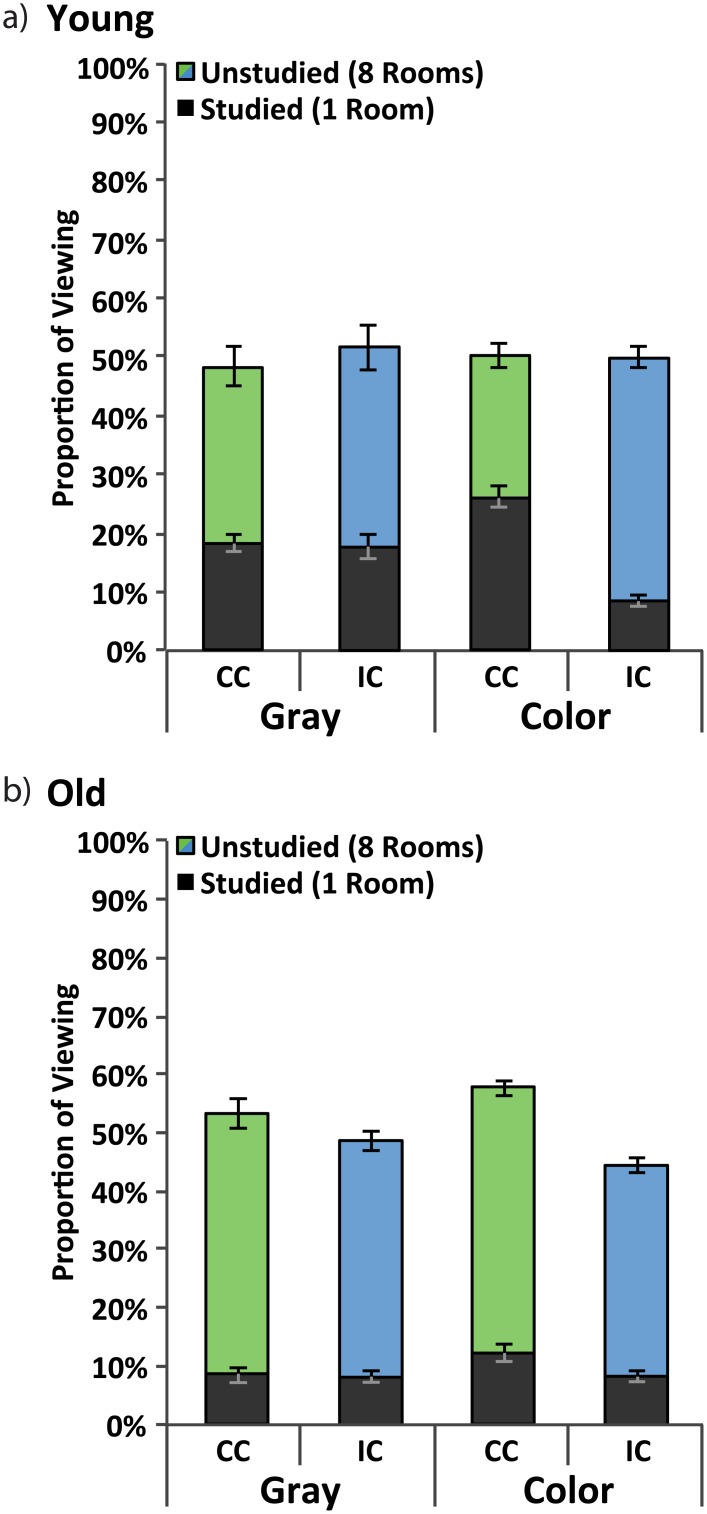
Eye-movement data. Proportion of viewing to rooms on the correct (green) and incorrect (blue) side of the building. Dark bars indicate face placements to the studied room on a given side whereas colored (green or blue) bars indicate face placements to any of the remaining eight unstudied rooms. Data are presented for both the Young Adult (a) and Older Adult (b) groups for both the gray building and color building viewing period; standard error bars are shown.

Young adults viewed the CC side of the building 48.3% and the CI side 51.7% of the time (Fig 7a); older adults viewed the CC side 52.3% and the CI side 47.7% of the time (Fig 7b). A Side (CC, CI) x Group (Young, Old) repeated measures ANOVA was performed and neither the main effect of side, *F*(1,38) = .03, *p =* .861, *η* ^2^ = .001, nor the interaction, *F*(1,38) = 1.31, *p =* .261, *η* ^2^ = .033, were significant. Thus participants directed a similar proportion of viewing to the CC and CI sides and this relationship was similar between the groups.

Proportion of viewing to each of the studied rooms was also calculated. For young adults, the proportion of viewing to each studied room was similar, 18.5% and 17.5%; paired-samples *t*(19) = .44, *p =* .666 (Fig 7a). For older adults, the proportion of viewing was also similar, 8.4% and 8.0%, *t*(19) = .45, *p =* .661 (Fig 7b). Viewing to CC-studied and CI-studied rooms was greater than chance (5.5%) for both groups (*p* < .05 in all cases). A Studied Room (CC-studied, CI-studied) x Group (Young, Old) repeated measures ANOVA was performed and neither the main effect of Studied Room, *F*(1,38) = .43, *p =* .518, *η* ^2^ = .011, nor the Interaction, *F*(38) = .08, *p =* .781, *η* ^2^ = .002, was significant. The main effect of Group, *F*(1,38) = 29.16, *p <* .001, *η* ^2^ = .434, was significant. Thus overall viewing to the studied rooms was less for older adults compared to younger adults, however, both groups did not disproportionately view either studied room during the gray building period. These data suggest that each face had been associated with two separate rooms and in the absence of contextual information, both rooms were equally viable alternatives.

#### Viewing During the Colored Building Context

Proportion of viewing to the CC and CI sides of the building were again computed when the colored building appeared. Young adults viewed the CC side of the building 50.3% of the time and the CI side 49.8%; this difference was not significant, *t*(19) = .16, *p =* .878 (Fig 7a). Older adults viewed the CC side of the building 56.5% and the CI side 43.6% of the time; this difference was significant, *t*(19)4.66, *p <* .001 (Fig 7a). A Side (CC, CI) x Group (Young, Old) repeated measures ANOVA showed a significant main effect of Side, *F*(1,38) = 10.00, *p =* .003, *η* ^2^ = .208, and a significant Interaction, *F*(1,38) = 8.86, *p =* .006, *η* ^2^ = .184. Thus, older adult participants distributed more viewing to the CC side of the building, while the young adult participants distributed their viewing evenly across the two sides.

Proportion of viewing to each of the studied rooms was also considered. Viewing to CC-studied and CI-studied rooms was greater than chance (5.5%) for both groups (*p* < .01 in all cases). Young adults looked at the CC-studied room 26.2% of the time and the CI-studied room 8.4% of the time; this difference was significant, *t*(19) = 7.46, *p <* .001 (Fig 7a). Older adults looked at the CC-studied room 12.0% of the time and the CI-studied room 8.1%; this difference was also significant, *t*(19) = 2.55, *p =* .019 (Fig 7b). A Trial Type (CC-studied, CI-studied) x Group (Young Adults, Old Adults) repeated measures ANOVA revealed a significant main effect of both Trial Type, *F*(1,38) = 58.86, *p <* .001, *η* ^*2*^ = .608, and Group, *F*(1,38) = 38.46, *p <* .001, *η* ^*2*^ = .503, as well as a significant Interaction, *F*(1,38) = 24.43, *p <* .001, *η* ^*2*^ = 391. These data demonstrate that participants in both groups preferred the CC-studied to the CI-studied room, but the difference was larger for younger adults.

#### Studied Room Preference

**Fig 8:**
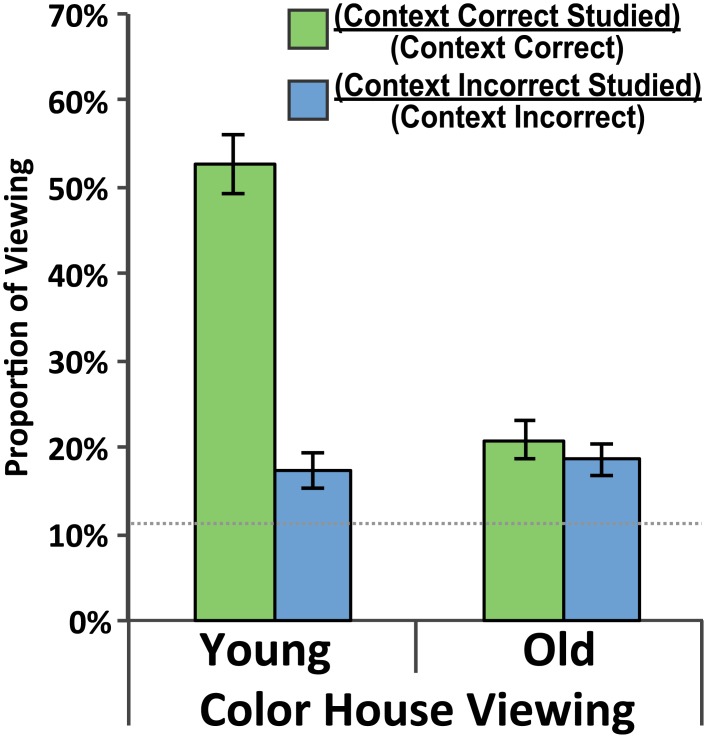
Studied room viewing preference. Preference for viewing of the studied room as a proportion of overall viewing to a given side of the building when the color building was presented. Green bars indicate a viewing preference for the studied room on the context-correct side of the building. Blue bars indicate a viewing preference for the studied room on the context-incorrect side of the building. Data are presented for both the Young Adult and Older Adult groups and standard error bars are shown. The dotted gray line indicates chance-level performance.

Preferential viewing to the studied room over other rooms on a given side of the building was considered when the colored building appeared (Fig 8). The proportion of studied room viewing to total viewing was computed for each side of the building. Young adult participants preferred the studied room 52.9% of the time on the CC side and 17.4% of the time on the CI side of the building. Older adult participants preferred the studied room 21.0% of the time on the CC side and 18.5% of the time on the CI side of the building. One-sample *t*-tests indicated that these studied-room preference were greater than chance (11.1%; *p <* .010 in all cases). A Side (CC, CI) x Group (Young, Old) repeated measures ANOVA was performed. The main effect of Side, *F*(1,38) = 49.53, *p <* .001, *η* ^*2*^ = .566, the main effect of Group, *F*(1,38) = 47.08, *p <* .001, *η* ^*2*^ = .553, and the Interaction, *F*(1,38) = 37.64, *p <* .001, *η* ^*2*^ = .498, were all significant. Post hoc paired-samples *t*-tests revealed the preference was greater for the CC side than the CI side for younger adults, *t*(19) = 7.22, *p <* .001, but the two were similar for older adults, *t*(19) = 1.10, *p =* .284. These data demonstrate that both groups preferred the studied room to other rooms on each side of the building and that the preference was stronger on the CC side, but only for young adults. Again these data suggest that participants showed a preference for the studied room even when they made a CI response.

#### Volumetric Measures

**Fig 9:**
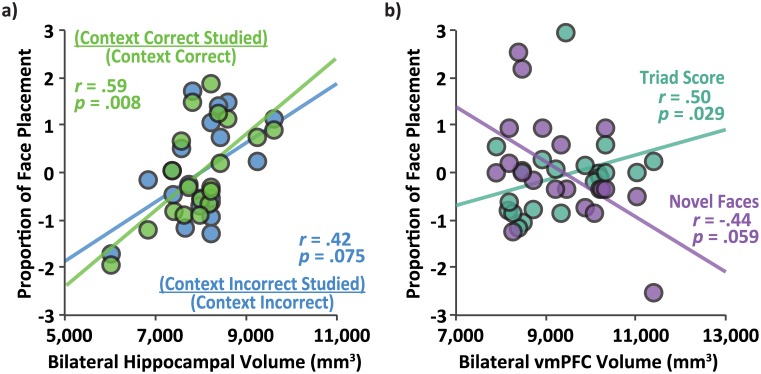
Behavior and volumetric relationships. *(*a) The correlation between bilateral hippocampal volume and preference for placing the face in the studied room as a proportion of all face placements to context-correct (green) and context-incorrect (blue) sides of the building. (b) The correlation between bilateral ventromedial prefrontal cortex volume and both correct novel face placements (purple) and Triad Scores (studied room placements on the context-incorrect side of the building divided by studied room placements to the context-correct side of the building; teal). All behavioral data were *z*-scored for presentation purposes.

Volumetric data were also acquired from cortical and subcortical brain structures for older adult participants. Given the vast literature summarizing hippocampal involvement in binding arbitrary relations among stimuli in a host of relational memory tasks (e.g., [27–34]), the relationship between hippocampal volume (controlling for cranial volume) and memory performance on this novel task was investigated. Controlling for age, hippocampal volume positively correlated with the proportion of responses to studied rooms (CC-studied and CI-studied combined), *r =* .56, *p =* .013. Bilateral hippocampal volume also positively correlated with the proportion of CC-studied rooms over all other rooms on the CC side of the building, *r =* .59, *p =* .008 (Fig 9a). There was a non-significant trend for the relationship between bilateral hippocampal volume and the proportion of CI-studied rooms over all other rooms on the CI side of the building, *r =* .42, *p =* .075 (Fig 9a). Taken together, these data demonstrate that older adults with larger hippocampi were better at selecting a studied room than those with smaller hippocampi.

Recent work has demonstrated the importance of vmPFC and vmPFC-hippocampal interactions in the formation of triadic memory representations [40,43]. The behavioral and eye-tracking data presented here are also consistent with the idea that participants formed room1-face-room2 triadic associations and further suggest that the two rooms may compete at the time of retrieval. In order to investigate the relationship between vmPFC volume (again controlling for cranial volume) and formation of triadic memory representations we calculated a Triad Score by computing the proportion of CI-studied room placements to CC-studied room placements for each participant. Participants who made lots of CI-studied placements had higher Triad scores indicative of greater competition between the two studied rooms. The correlation between vmPFC volume and the Triad Score was significant, *r =* .50, *p =* .029 (Fig 9b). Thus, participants with larger vmPFC volumes appear to weigh learned associative information more heavily than contextual-rule information. In fact, the correlation between CC novel face placement (i.e., no associative information) and vmPFC approached significance, *r = -*.44, *p =* .059 (Fig 9b), suggesting that participants with large vmPFC volumes undervalued the contextual-rule structure.

#### Rule vs. Triad

The volumetric data suggest that those participants with larger Triad Scores are less likely to depend on the contextual rule-structure. To directly evaluate this relationship the correlation (one-tailed tests) between CC novel face placements (i.e., pure measure of rule learning) and Triad Scores were calculated for both younger and older adults. Consistent with this hypothesis, negative correlations were significant both for young (*r* = -.83, *p* < .001) and older (*r* = -.42, *p* = .034) adults. Furthermore, Fisher’s *r*-to-*z* transformation was calculated and statistically compared [53]; these correlations were significantly different from one another (*z* = 2.13, *p* = .033 2-tailed) suggesting that the relationship was stronger for young compared to older adults.

#### Summary

As in Experiment 1, participants consistently preferred the CC side of the building when making their responses. While the studied room was preferred over other rooms on each side of the building, participants still selected the CC-studied room more frequently than the CI-studied room. This was true of both young adults and older adults, however, both the preference for the studied room generally and the preference for the CC-studied room over the CI-studied room was reduced for older adults. As in Experiment 1, these data are consistent with the idea that participants are able to form room1-face-room2 triadic representations and that those studied rooms compete at test time. Viewing behavior during study leant further support for this suggestion. Over multiple encoding opportunities, participants viewed not only the CC-studied room (which was on the screen throughout the study trial), but also the CI-studied room. CI-studied room viewing increased across encoding repetitions, again suggesting that a single face had been associated with two rooms. At test, when provided with only a contextually-ambiguous stimulus (i.e., gray building), participants viewed both studied rooms equally (i.e., the triadic representation was activated) suggesting competition between the two learned target locations. Once building context was revealed, conflict was resolved and participants were able to correctly identify the CC-studied room and respond appropriately. However, the CC-studied room preference was greater for young adults, suggesting an impaired ability to incorporate the contextual information and select the CC-studied room among older adults.

These data suggest that while the older adults were able to learn face-room associations in this task, they were less likely to adequately incorporate contextual information to accurately dissociate overlapping memory representations. Hippocampal volume positively correlated with CC-studied room and CI-studied room preference as well as the overall proportion of CC-studied and CI-studied face placements. Thus, those participants who are more likely to select a studied room have relatively larger hippocampi compared to participants who are less likely to remember where a face was studied. These data support previous findings that the hippocampus supports high-resolution, object-location bindings, and relational memory (cf. [6,15]). Finally, Zeithamova and colleagues [40] have reported that together with the hippocampus, the vmPFC is essential for constructing distinct memory representations into an integrated representation that can be used adaptably in service of memory performance. In this sample, vmPFC volume positively correlated with our Triad metric, further highlighting a role for vmPFC in successful binding of multiple distinct representations into a single flexible associative memory representation.

## General Discussion

Across two experiments, participants demonstrated the ability to combine associative information (face-room pairings) with contextual cues (gender-by-side rule) to guide behavior. Successful associative learning was evident in participants’ ability to consistently place faces in studied rooms—whether the context was correct or not. Successful contextual learning was evident in participants’ tendency to use the CC side for both novel and previously studied faces. Eye tracking during a contextually ambiguous preview period corroborated both of these findings, participants preferentially viewed rooms in which the studied face had previously appeared, and shifted their gaze toward the CC response when the context was revealed.

There are at least two possible interpretations as to why more CC than CI responding occurs in this task. One possibility is that participants are learning something about the underlying gender-by-side rule structure and are using that information to help them decide where to place a given face. Alternatively, perhaps participants are simply forming imprecise representations of the individual face-room associations and CC responses are simply the result of CC-studied room near misses. We believe that our data support the former interpretation. In Experiment 1, the proportion of incorrect face-placements was calculated for trials in which the studied room was in one of the two center columns (i.e., adjacent to the gender-by-side rule boundary). If responding was the result of imprecise memory representations resulting in a near-miss of the studied room, one would expect participants to be equally likely to make an adjacent error to the CC or CI sides of the studied room. However, participants were significantly more likely to respond on the CC side of the building. Furthermore, in Experiment 2, participants were asked to make choices about novel faces for which no face-room associations exist. Again, participants were more likely to make CC than CI responses. Therefore, we believe that these data suggest that participants learned at least partial rule information. Further neuroimaging research is necessary to determine the underlying neural correlates of contextual-rule-learning in this task.

Furthermore, the pattern of participants’ errors showed more complex trade-offs between context and association than would be suggested by their accuracy alone. In Experiment 1, repeated exposure to the association resulted in increased CC-studied responses, but also dramatically increased CI-studied responses, resulting in a slight *decrease* in CC-nonstudied responses overall. This shows an interesting asymmetry between associative and contextual information: all of the repeated face-room pairings were CC, and yet the net result was to increase the importance of specific associations at the cost of incorporating contextual information.

In Experiment 2, younger and older adults’ responses and viewing behavior showed a preference for the studied rooms, and younger adults showed an even stronger preference for CC responding than in Experiment 1. However, older adults showed weak preferences for CC responses, which had the predictable effect of increasing their tendency to choose a CI studied room. These findings highlight the interplay of competing associative representations, and the memory systems that underlie them.

Volumetric data from our older adult participants provide initial evidence for candidate brain systems supporting associative memory in this task. These data suggest that, for older adults, successful memory for face-room associations correlated with hippocampal volume. This was true for both CC and CI face-room associations, although the relationship was numerically larger for CC associations. While the hippocampus has a long history of supporting associative/relational memory (e.g., [27–34]), other neural regions also play an active role in successful associative memory formation and use. Recent research has proposed that one such region, the vmPFC, plays a very specific role in memory (for review see [42]). For example, Preston and Eichenbaum [42] have suggested that while the hippocampus binds elements into new associations, when information between learned associations overlaps, the vmPFC plays a critical role in binding these related associations together into a single triadic representation. In the task here, we propose that the vmPFC combines overlapping learned face-room associations into a single representation in which a single face is associated with multiple contexts. Whereas triadic associations play a facilitatory role in, for example, the transitive inference task [40,43]; in the current task, such associations appear to foster competition between the two component pairwise associations. Interestingly, participants with larger Triad scores were less likely to demonstrate learning of the gender-by-side rule. Therefore, in this task, it appears that formation of triadic associations conflicted with overall rule-learning.

Indeed, we found that participants with larger vmPFC volume were more likely to create multi-representation triadic associations; and participants with smaller vmPFC volumes did not form such associations and thus relied more heavily on contextual information. Thus, while vmPFC has been highlighted as essential in successful performance on transitive inference tests [40,43], these data demonstrate, in a different paradigm, the importance of this region in binding disparate, but overlapping information.

Across both experiments, participants disproportionately selected the CI-studied room over all other unstudied rooms. This tendency is somewhat unintuitive. Our interpretation is that participants do not enjoy the experimenter’s knowledge of the contextual rules used to generate the face-room pairings, but rather had to deduce them from the observed face-building pairings. Thus participants did not represent the contextual structure as fixed, binary rules, but rather as statistical regularities of the task. This statistical representation of general tendencies must compete with memory representations of specific associations, both contributing to generating behavioral responses in the large search space of the building.

This suggests that rather than providing clear response constraints in situations with an ambiguous target response, contextual rules help weight responses rather than define them (cf. [45,54]). This idea is consistent with the parallel-task set model (e.g., [21]). On a given trial, multiple task-sets (cf. [55]) are activated (in the exclude recognition framework where this model was developed, one task-set is based on contextual familiarity and the other on associative recognition). Both task-sets include information about the memory representation, the appropriate response, and a motor plan to execute the response, and both operate in parallel. When task-sets point to separate outcomes and neither set is aborted, the faster response wins. This suggests that in our design, the CI-studied response is more rapidly available than the contextual rule-based response, and inhibitory executive control processes may be required to integrate the slower CC response [21,22]. Further investigation is necessary to explore this hypothesis. The weaker preference for CC responses among older adults is suggestive in this case, since a body of literature suggests that older adults may have difficulty inhibiting and integrating interfering memory responses [56–58]. Further neuroimaging investigations using additional imaging techniques (e.g., fMRI, DTI, etc.) are necessary to both identify neural correlates of the contextual-rule learning in this task as well as to understand the precise dynamics of these competing and cooperating systems.

All together, these data argue for a nuanced view of the interaction between context and associative information. Within this novel context-guided retrieval paradigm, the overlapping associative information is dissociable only by incorporating contextual information. The correct behavioral response depends on distinguishing and correctly employing these pieces of information. Interference between these information sources results in the selection of an incorrect response, but this process fails gracefully, selecting either a “next best” competing associate, or guiding a response consistent with the experiment’s contextual structure. The age-related performance declines we observed, likely related to changes in brain structures across the lifespan, tended to shift behavior toward these second-best responses based on only the associative memory system rather than an integration of representations from both the associative and contextual-rule learning system. We interpret this decline as a breakdown in coordination between brain systems wherein a single source of information becomes dominant.

## Supporting Information

S1 Table All individual subject data contributing to the means reported in this report are available in the supplementary spreadsheet. (XLSX)
